# Development and Validation of a Fetal Cardiovascular Disease Severity Scale

**DOI:** 10.1007/s00246-014-0911-9

**Published:** 2014-05-07

**Authors:** Brooke T. Davey, Mary T. Donofrio, Anita J. Moon-Grady, Carlen G. Fifer, Bettina F. Cuneo, Christine B. Falkensammer, Anita L. Szwast, Jack Rychik

**Affiliations:** 1 Connecticut Children’s Medical Center, Hartford, CT USA; 2 The Children’s Hospital of Philadelphia, 34th Street and Civic Center Blvd., Philadelphia, PA 19104 USA; 3 Children’s National Medical Center, Washington, DC USA; 4 University of California - San Francisco, San Francisco, CA USA; 5 University of Michigan, C. S. Mott Children’s Hospital, Ann Arbor, MI USA; 6 Children’s Hospital Colorado, University of Colorado School of Medicine, Denver, CO USA

**Keywords:** Severity, Congenital heart disease, Scale, Fetal echocardiography, Cardiac anomaly

## Abstract

Prenatal heart disease spans the spectrum of severity from very mild to severe life-threatening conditions. An accepted scale for grading fetal cardiovascular disease severity would aid in anomaly standardization, counseling, and future research. The Fetal Cardiovascular Disease Severity Scale with seven severity grades ranging from mild (grade 1) to severe (grade 7) disease was developed. Severity grade relates to the cardiovascular condition diagnosed by fetal echocardiography, with factors including postnatal intervention, number of interventions anticipated, likelihood of two-ventricle repair versus single-ventricle palliation, and overall prognosis. A survey describing 25 cardiac anomalies was offered to fetal cardiologists at six institutions for validation of scale reliability among practitioners. The study participants graded defects using this scale. A smaller group graded anomalies again more than 2 weeks after the initial survey. The intraclass correlation coefficient (ICC) was used to assess agreement of the respondents. The survey participants were 14 experienced fetal cardiologists: 9 from the Children’s Hospital of Philadelphia (CHOP) and 5 from five additional institutions in the United States. The initial survey ICC was high [0.93; 95 % confidence interval (CI) 0.88–0.96]. The subanalysis showed a higher ICC for the participants outside CHOP (0.95; 95 % CI 0.91–0.98 vs. 0.92; 95 % CI 0.86–0.96, respectively). The ICCs were high for all the fetal cardiologists participating in the repeat evaluation, ranging from 0.92 to 0.99 (95 % CI 0.65–1.00). The Fetal Cardiovascular Disease Severity Scale demonstrated good inter- and intrarater reliability among experienced fetal cardiologists and is a valid tool for standardization of prenatal cardiac diagnostic assessment across institutions. The scale has applications for parental counseling and research in fetal cardiovascular disease.

## Introduction

Judgment of the severity of a particular form of congenital heart disease (CHD) comprises a multitude of factors including the anatomy and pathophysiology of the lesion as well as the number and type of interventions available to treat or palliate the condition. The outcomes and overall quality of life created through surgical and medical management, both short and long term, also are factors that enter into consideration of severity [1, 9, 10].

With the advent of fetal echocardiography, diagnosis of CHD before birth currently is well established [4, 5]. Early diagnosis enables a smooth transition from pre- to postnatal life and improves infant survival and morbidity [2, 7, 11]. The anatomy of the lesion, its implications for delivery, expected neonatal and life-long interventions, and overall prognosis all are discussed currently with families before the birth of a baby with CHD [8].

To provide accurate estimates of the clinical course of CHD based on fetal imaging alone, prenatal cardiac care providers, including pediatric cardiologists, maternal fetal medicine specialists, genetic counselors, and nurses, must convey an overall assessment of the lesion severity to families during counseling and to the medical team caring for the baby during delivery and after birth.

A comprehensive, standardized, and validated system to rate the severity of CHD in the fetus would be of great value to patients, their families, and the medical community caring for them. A uniform measure of disease severity could improve communication and conveyance of information at prenatal counseling. It also would aid in research studies assessing outcomes, particularly across institutions.

This study aimed to introduce such a tool, the Fetal Cardiovascular Disease Severity Scale, and to describe its validation across different centers.

## Methods

### Design of the Scale

Experts in the field of fetal and pediatric cardiology developed the Fetal Cardiovascular Disease Severity Scale to grade the severity of CHD (Table 1). In designing this scale, the experts carefully considered those elements that come into play in the attempt to describe the severity of a particular form of CHD at the time of fetal diagnosis and counseling.

**Table 1:** Fetal cardiovascular disease severity scale

Status	Definition	Treatment	Prognosis/anticipated outcome	Brief summary description	2V	1V
Level 1	Cardiovascular finding with minimal, if any, negative impact on well-being	None	Excellent/normal quality of life	No significant disease	✓	✗
Level 2	Cardiovascular abnormality for which treatment *may* be necessary. Postnatal follow-up is necessary	Medical management may be required in utero or after birth. Surgery or catheter therapy is *possible* but will need to await postnatal assessment to be certain	Excellent/normal quality of life	Mild disease, need for intervention (surgery/catheter) possible	✓	✗
Level 3	Cardiovascular abnormality/*simple* form of CHD (two-ventricle)	Surgery or catheter therapy will, with *certainty,* be required	Excellent/normal quality of life	One intervention (surgery/catheter) necessary with excellent outcome	✓	✗
Level 4	Cardiovascular abnormality/*complex* form of CHD (two-ventricle)	Surgery will, with *certainty*, be required. Further additional interventions or surgery *may* be necessary at some point in life	Good/close to normal quality of life	One intervention necessary, multiple; interventions (surgery/catheter) are possible during lifetime; good outcome	✓	✗
Level 5	Cardiovascular abnormality/*complex* form of CHD, single- or two-ventricle type	Fontan surgical palliation strategy is required for single-ventricle patients, or surgery for two-ventricle repair will, with *certainty*, be required, and further intervention or surgery will, with *certainty*, be necessary in the future	Prognosis is fair to good; infant is likely to survive surgery. Quality of life *may* be impaired or duration of life *may* be limited	Single-ventricle strategy, low risk; two-ventricle strategy, with definite need for additional procedures during lifetime; fair-to-good overall outcome	✓	✓
Level 6	Cardiovascular abnormality/*complex* form of CHD, single- or two-ventricle type	Fontan surgical palliation strategy is required, but at high risk, or two-ventricle repair, but at high risk	Prognosis is poor to fair; risk of death is possible; and long-term complications are highly likely. Survival beyond childhood is poor.	Single-ventricle strategy, high risk; two-ventricle strategy with variable outcome and limited life expectancy	✓	✓
Level 7	Cardiovascular abnormality/*complex* form of CHD with very poor prognosis	Intervention may be offered, but the expected outcome is poor	Fetal or perinatal demise likely, despite intervention	Poor outcome; survival beyond early period of life not expected	✓	✓

Scale composed of 7 grades, italicized important considerations for an overall assessment of lesion severity

*1V* single ventricle palliation anticipated, *2V* biventricular repair anticipated

To provide adequate gradation of each lesion with its own unique constellation of features, seven levels of severity were included in the scale. It then was sent for evaluation and review to the group of cardiologists, who further modified and revised it based on their feedback until a consensus was reached as to the content and design of the scale.

The degree of severity in the scale is determined by elements including the general category of anatomic complexity of heart disease (e.g., isolated valve pathology vs. isolated septal defect vs. complex outflow tract abnormality vs. ventricular hypoplasia), the need for postnatal intervention and its complexity, the number of lifelong interventions (catheter based or operative) anticipated, the likelihood that an intervention will lead to a two-ventricle repair versus single-ventricle palliation, and the overall prognosis with regard to quality and duration of life.

Table 1 describes the seven levels of severity ranging from level 1 (least severe) to level 7 (most severe). Each level is structured by a definition concerning anatomic categorization, the treatment anticipated, and the prognosis or anticipated outcome. A brief summary term describing each level is listed. Subjects with a possible two-ventricle management strategy can be graded at levels 1–7, whereas those with a single ventricle are graded only at levels 5–7.

Genetic/chromosomal abnormalities and extracardiac anomalies are not included in the scale because they may independently affect the prognosis irrespective of the cardiac condition. They are therefore considered additive to the cardiovascular severity. In addition, the Fetal Cardiovascular Disease Severity Scale is designed to grade the severity of structural CHD. Cardiomyopathies and arrhythmias are excluded because they have their own unique considerations and features that influence severity and because the aim was to create a more streamlined assessment tailored to the gradation of structural cardiac malformations.

### Validation of the Scale

This assessment aimed to validate the Fetal Cardiovascular Disease Severity Scale and to determine its use among fetal cardiologists. Validity was determined by the degree of agreement among experienced fetal cardiologists in grading a sample of cases.

A survey was administered to fetal cardiologists within our institution, the Children’s Hospital of Philadelphia (CHOP), and to experienced fetal cardiologists at other institutions across the United States. Input provided by fetal cardiologists from multiple different centers introduced different institutional experiences, regional biases, and local outcomes to this analysis. The participating centers included the Children’s National Medical Center (MTD, Washington DC, USA), the University of California–San Francisco (AMG; San Francisco, CA, USA), the CS Mott Children’s Hospital at the University of Michigan (CGF; Ann Arbor, MI, USA); and the Advocate Children’s Hospital (BC; Oak Lawn, IL, USA). One of the fetal cardiologists at CHOP (C.B.F.), who had recently arrived from another institution [Texas Children’s Hospital (TCH)], was therefore considered to offer judgments that reflected her experiences in fetal cardiac care at TCH and not CHOP.

The survey assessment was sent via email to all the study participants. The survey consisted of 25 hypothetical case examples covering a wide spectrum of cardiovascular conditions diagnosed in fetal life by echocardiography (Table 2). The participants were asked to grade each of the lesions using the scale configured in Table 1. The responses then were emailed back to CHOP for compilation of data. The fetal cardiologists were blinded to the answers of the other participants.

**Table 2:** Survey of 25 case descriptions

Case no.	Disease description	Grade
1	TOF, mild pulmonic stenosis	
2	Echo bright spot on left ventricular papillary muscles, otherwise normal heart structure and function	
3	HLHS, mitral atresia, aortic atresia, open unrestrictive atrial septum, no tricuspid regurgitation	
4	Midmuscular VSD, small	
5	Complete AV canal defect, mild AV valve regurgitation	
6	Left ventricle-to-right ventricle size discrepancy, left superior vena cava to coronary sinus, mild aortic arch narrowing	
7	Heterotaxy syndrome, single ventricle, pulmonary atresia, total anomalous pulmonary venous return (infradiaphragmatic)	
8	TGA (intact ventricular septum)	
9	Two-vessel umbilical cord, otherwise normal heart structure and function	
10	Double-outlet right ventricle, subaortic VSD with severe pulmonic stenosis	
11	HLHS, mitral stenosis, aortic atresia, intact atrial septum	
12	Floppy, redundant atrial septum and premature atrial contractions, otherwise normal heart structure and function	
13	TOF with pulmonary atresia, very small hypoplastic branch pulmonary arteries, and suspicion of multiple aortopulmonary collaterals	
14	Truncus arteriosus (type 2A with VSD and branch pulmonary arteries arising from side of trunk), no truncal valve stenosis or regurgitation	
15	Large perimembranous VSD	
16	Tricuspid atresia, normally related great vessels, moderate size VSD, moderate pulmonic stenosis	
17	Pulmonary atresia with intact ventricular septum, marked right ventricular hypoplasia, severe tricuspid valve hypoplasia; inflow into the ventricle noted, but no tricuspid regurgitation seen	
18	Truncus arteriosus (type 1A with VSD and main pulmonary artery segment giving rise to well-formed branch pulmonary arteries) abnormal truncal valve with severe truncal insufficiency	
19	TGA, VSD, pulmonic stenosis	
20	Ebstein’s anomaly, pulmonary atresia, severe tricuspid regurgitation, severe hydrops	
21	Complete AV canal defect, balanced, no AV valve regurgitation	
22	Interrupted aortic arch type B, VSD, mild subaortic narrowing	
23	Critical aortic valve stenosis, normal size and functioning left ventricle	
24	Pulmonary atresia with intact ventricular septum, near normal size tricuspid valve, plate-like pulmonary atresia, moderate tricuspid regurgitation	
25	Coarctation of the aorta, normal left ventricle	

Fetal cardiologists were asked to assess each case and assign a severity grade from 1 to 7 to the case using the Fetal Cardiovascular Disease Severity Scale

*TOF* tetralogy of Fallot, *HLHS* hypoplastic left heart syndrome, *AV* atrioventricular, *VSD* ventricular septal defect, *TGA* transposition of the great arteries

### Data Analysis

The intraclass correlation coefficient (ICC) was used to assess agreement between respondents to determine interrater reliability. The minimum, maximum, range, standard deviation, and interquartile ranges of the case severity grades from the respondents also were computed. Next, the 25 cases on the worksheet were shuffled to change their order and then sent to a smaller subgroup of the initial participants, including all nine CHOP fetal cardiologists and one outside fetal cardiologist. These participants, blinded to their previous answers, were again asked to grade the diagnoses using the severity scale. The ICC comparing the answers of the individual respondent with the same diagnosis at different points in time was used to assess for agreement and to determine the intrarater reliability of the scale.

## Results

The participants in the survey evaluation were 14 physicians: 9 fetal cardiologists trained in fetal cardiology and/or practicing it at the Fetal Heart Program at CHOP and 5 fetal cardiologists trained in fetal cardiology and/or practicing it at other institutions. Of these 14 participants, 10 evaluated the severity of the same diagnoses on a shuffled worksheet, with a minimum of 2 weeks between the completion of the two surveys. The ICC of 0.93 [95 % confidence interval (CI) 0.88–0.96] among all the respondents demonstrated a high level of agreement between providers. The fetal cardiologists from outside institutions demonstrated an ICC of 0.95 (95 % CI 0.91–0.98), whereas the ICC for the participants at CHOP was 0.92 (95 % CI 0.86–0.96).

The range of severity grade responses by case is listed in Table 3. Overall, 60 % of the cases had respondents who offered a severity scale grade within one or two severity levels of each other, and 96 % of the cases had a severity scale grade within three levels of each other or less. In one case, the participants demonstrated a severity grade range of four levels (case 23: critical aortic valve stenosis, normal size, and functioning left ventricle).

**Table 3:** Initial survey results of severity level assessment by case

Case no.	Case severity level min–max (range)	SD
1	3–4 (2)	±0.4
2	1 (1)	±0
3	5–6 (2)	±0.5
4	1–2 (2)	±0.5
5	3–5 (3)	±0.6
6	1–3 (3)	±0.4
7	5–7 (3)	±0.6
8	3–5 (3)	±0.5
9	1 (1)	±0
10	3–5 (3)	±0.6
11	6–7 (2)	±0.5
12	1–2 (2)	±0.4
13	5–6 (2)	±0.5
14	4–5 (2)	±0.5
15	2–3 (2)	±0.4
16	5 (1)	±0
17	5–6 (2)	±0.5
18	5–7 (3)	±0.6
19	4–6 (3)	±0.6
20	7 (1)	±0
21	3–5 (3)	±0.6
22	4–5 (2)	±0.5
23	3–6 (4)	±0.7
24	3–5 (3)	±0.6
25	3–4 (2)	±0.5

Minimum, maximum, range, and SD of severity grade assignments are listed by case

*Min* minimum, *max* maximum, *SD* standard deviation

**Fig. 1:**
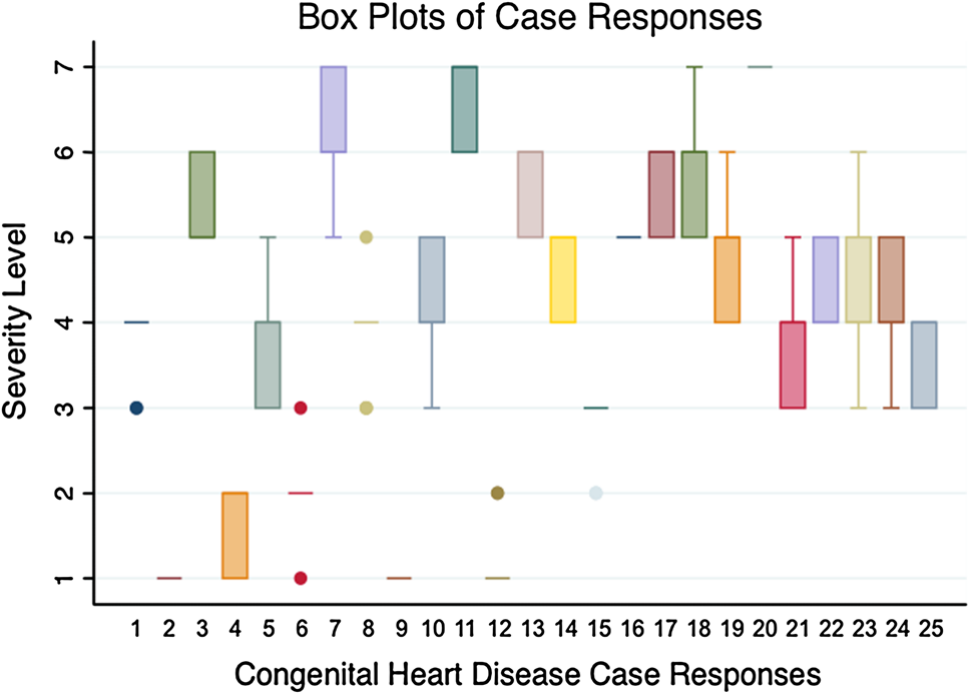
Boxes and horizontal single lines represent the interquartile range of responses to assignment of severity level by case, whereas whiskers and dots represent outlying responses. For example, in case 2, all the participants agreed that the case should be assigned a severity level of 1, represented by the single line. In case 8, all the participants within the interquartile range assigned the case a severity level of 4, again represented by the single line. The dots represent the outliers who graded the case at a severity level of 3 or 5. Finally, in case 21, the participants within the interquartile range assigned the case a severity level of either 3 or 4, represented by the rectangle. An outlier assigned it a severity level of 5, represented by the whisker. This figure demonstrates that interquartile ranges for all the cases were within one or two severity levels, whereas all the outliers were within one level of the interquartile range

All the cases had an interquartile range within one or two levels of severity, indicating that responses outside these levels were outliers (Fig. 1). The ICCs were high for all the fetal cardiologists participating in the repeat evaluation, ranging from 0.92 to 0.99 (95 % CI 0.65–1.00) (Table 4).

**Table 4:** Survey results of intraobserver reliability assessment by provider

Provider	ICC	95 % CI for ICC
1	0.97	0.93–0.99
2	0.99	0.98–1.00
3	0.99	0.97–0.99
4	0.95	0.89–0.98
5	0.92	0.65–0.97
6	0.94	0.86–0.97
7	0.99	0.97–0.99
8	0.97	0.93–0.99
9	0.97	0.93–0.99
10	0.95	0.89–0.98

ICCs with 95 % CIs for each provider were used to assess intraobserver variability

*ICC* intraclass correlation coefficient, *CI* confidence interval

## Discussion

Currently, the first point of entry into the realm of congenital heart care commonly occurs before birth with a prenatal diagnosis. Establishment of disease severity among the myriad of conditions that exist is an essential aspect of prenatal care for CHD. Disease severity influences decisions concerning continuation of the pregnancy, dictates management for the remainder of gestation, and affects planning for delivery and postnatal care. A commonly accepted grading system for fetal cardiovascular conditions among practitioners would be of great value in creating a common language, and importantly, could function as an instrument for investigational studies. A means of categorizing fetal cardiovascular disease severity, in essence the diagnosis of CHD at its earliest presentation, also could serve as a tool for gauging resource use and cost effectiveness. In this report, we describe the development of such a tool and its validation.

Methods for categorizing the clinical variability in congenital heart anomalies currently exist, but none are specifically designed for prenatal use. Task Force One of the 32nd Bethesda Conference of the American College of Cardiology uses elements of both anatomic diagnosis and surgical repair to divide adult patients with congenital heart defects into three groups of severity [12]: simple CHD, CHD of moderate severity, and CHD of great complexity, also described as simple, moderate, and severe CHD.

The Aristotle Score for Congenital Heart Surgery is designed to rate the complexity of surgical procedures used in palliating CHD based on morbidity, mortality, and anticipated level of surgical difficulty [6]. Patient characteristics also are taken into consideration with this system to provide a comprehensive score.

A consensus-based method of risk adjustment for in-hospital mortality after surgery to repair CHD is called the Risk Adjustment for Congenital Heart Surgery (RACHS-1) [3]. This system is designed to assess perioperative surgical mortality for children with CHD who are 18 years old or younger and allows for meaningful comparisons of surgical outcomes across institutions.

The aforementioned scoring systems do not provide a framework for a comprehensive assessment of CHD severity diagnosed in fetuses during the prenatal period, whereas the Fetal Cardiovascular Disease Severity Scale is uniquely designed for this patient population. In addition, these scoring systems take a more simplified approach to grading a group of extremely diverse cardiac lesions with unique features that affect risk and outcome. The 7-point system of the Fetal Cardiovascular Disease Severity Scale takes into account features beyond the anatomic complexity of the lesion, including need and number of interventions, two- versus single-ventricle palliation, and overall prognosis, making this scale a more comprehensive assessment of severity.

High ICCs comparing responses of fetal cardiologists with one another and with themselves indicate that the Fetal Cardiovascular Disease Severity Scale is a valid method for assessing structural CHD diagnosed in prenatal life. Agreement between the participants at CHOP (ICC 0.92) was slightly less than between those at the outside institutions (ICC 0.95). This may have been due to a higher number of CHOP participants from CHOP (9 participants) than from outside institutions (5 participants), leading to more opportunity for variability in response grades.

The ranges for the responses typically fell between one and two grades of severity, with some cases involving three grades, although the interquartile ranges all were within one or two levels. The sole case with a range of four severity grades had “critical aortic valve stenosis, normal size, and functioning left ventricle.” Of the 14 respondents, 12 identified this diagnosis as falling within severity grade level 4 or 5. One respondent graded the severity in this case as level 3, whereas one other respondent graded it as level 6. However, during the second evaluation to assess intrarater reliability, both respondents, blinded to their previous answers, independently graded the severity of the same diagnosis as level 4.

The description of critical aortic valve stenosis provided to the participants did not identify the gestational age of the fetus. This could have influenced the grade assignment because critical aortic stenosis with normal left ventricular function in a fetus at 20 weeks could potentially progress in utero, leading to a newborn with poor ventricular function or raising concern about the development of hypoplastic left heart syndrome. Such a diagnosis made at 36 weeks gestation would be less likely to create uncertainty about progression of the disease.

Certain conditions, such as the aforementioned case of critical aortic stenosis, can evolve anatomically during gestation. In other cases, the anatomy can lead to hemodynamic compromise, as in dysplastic tricuspid valve with tricuspid valve regurgitation, in which the degree of regurgitation worsens during gestation, causing hydrops and affecting prognosis.

The grading system used in the Fetal Cardiovascular Disease Severity Scale can be applied to each individual at multiple points in gestation as the fetal cardiovascular disease evolves. This can provide families with a more concrete parameter for understanding the implication of these changes during gestation. In addition, it can give fetal cardiologists a way to communicate the fetal cardiovascular disease course during gestation effectively to obstetricians and postnatal cardiologists.

In general, variability in responses across participants may be influenced by either institutional or personal experience with a specific individual defect. Furthermore, particular conditions in fetal life may be difficult to grade because they are plagued by uncertainty in outcomes based on a particular strategy, such as the notion that the outcome of a “good” single-ventricle strategy may be superior to that of a “poor” two-ventricle repair strategy. Nevertheless, our analysis showed that for a large heterogeneous group of 25 anomalies, the Fetal Cardiovascular Disease Severity Scale demonstrated excellent interclass correlation and hence can be used between practitioners and across institutions.

This assessment had several limitations. The survey was a subjective evaluation by a limited number of attending fetal cardiologists with variable years of practitioner training and experience. It did not include the gestational age of the fetuses, which could have influenced the grade assignment. Responses could have varied due to recall bias of the most recent or more memorable cases. The scale was assessed by fetal cardiologists and has not been validated among maternal-fetal medicine specialists or other practitioners who may care for patients with prenatal CHD. In addition, the scale did not take into account genetic information or the presence of other extracardiac anomalies that could independently affect long-term outcome and prognosis.

Finally, the scale was designed for a general global assessment of CHD severity. Therefore, factors that contribute to severity (e.g., the number of interventions anticipated and overall prognosis) are paired within the scale. It is possible that for some conditions, the number of interventions or the complexity of the intervention and prognosis may be disparate. Hence, a particular diagnosis may not fit precisely within one level, which may contribute to variability in grades.

The Fetal Cardiovascular Disease Severity Scale demonstrated good inter- and intrarater reliability among experienced fetal cardiologists. It therefore is a valid tool for standardization of prenatal prognostic assessment of CHD severity across institutions. It also can be used at different points in time throughout gestation as a marker of disease evolution. This scale can be used as a standard component of data collection for research and has applications for parental counseling and transition of care during delivery and after birth. Further evaluation of this scale may be applied prospectively to patients with CHD diagnoses made in fetal life and validated on the basis of actual outcomes, including number of interventions and quality-of-life measures.
